# A global non-coding RNA system modulates fission yeast protein levels in response to stress

**DOI:** 10.1038/ncomms4947

**Published:** 2014-05-23

**Authors:** Hui Sun Leong, Keren Dawson, Chris Wirth, Yaoyong Li, Yvonne Connolly, Duncan L. Smith, Caroline R. M. Wilkinson, Crispin J. Miller

**Affiliations:** 1 Applied Computational Biology and Bioinformatics Group, Cancer Research UK Manchester Institute, University of Manchester, Wilmslow Road, Manchester M20 4BX, UK; 2 Biological Mass Spectrometry Facility, Cancer Research UK Manchester Institute, University of Manchester, Wilmslow Road, Manchester M20 4BX, UK; 3 Cell Regulation Group, Cancer Research UK Manchester Institute, University of Manchester, Wilmslow Road, Manchester M20 4BX, UK; 4 These authors contributed equally to this work

## Abstract

Non-coding RNAs (ncRNAs) are frequent and prevalent across the taxa. Although individual non-coding loci have been assigned a function, most are uncharacterized. Their global biological significance is unproven and remains controversial. Here we investigate the role played by ncRNAs in the stress response of *Schizosaccharomyces pombe*. We integrate global proteomics and RNA sequencing data to identify a systematic programme in which elevated antisense RNA arising both from ncRNAs and from 3′-overlapping convergent gene pairs is directly associated with substantial reductions in protein levels throughout the genome. We describe an extensive array of ncRNAs with *trans* associations that have the potential to influence multiple pathways. Deletion of one such locus reduces levels of *atf1*, a transcription factor downstream of the stress-activated mitogen-activated protein kinase (MAPK) pathway, and alters sensitivity to oxidative stress. These non-coding transcripts therefore regulate specific stress responses, adding unanticipated information-processing capacity to the MAPK signalling system.

## Abstract (web summary)

Non-coding RNAs are widely expressed, yet their functions remain poorly understood. Here, Leong *et al*. identify a set of antisense RNAs elevated during the yeast stress response that directly correlate with reduced protein levels, indicating a general regulatory effect of antisense expression.

## Graphical abstract

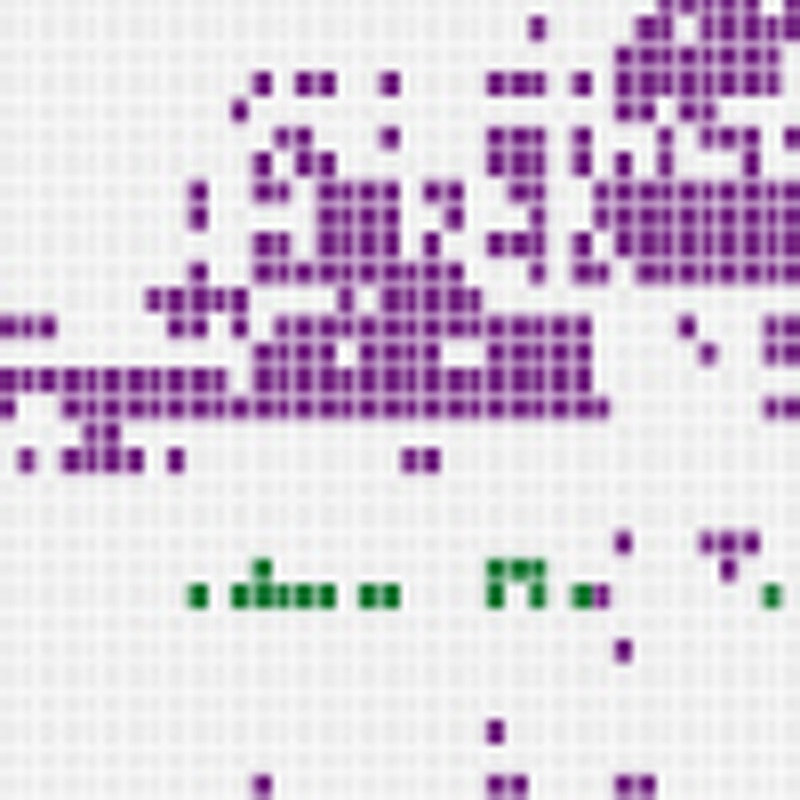

Mitogen-activated protein kinase (MAPK) cascades transduce information arising from events external to the cell, such as environmental stresses through the successive phosphorylation of a sequence of protein kinases, and the consequent phosphorylation of a variety of downstream effectors, including transcription factors. The fission yeast stress-activated MAP kinase (SAPK) pathway is conserved with the p38 and JNK pathways in humans, and comprises the MAPKKKs Win1, Wis4, the MAPKK Wis1 and the MAPK Sty1 (ref. 1). The main downstream effector of Sty1 is Atf1, an ATF/CREB family transcription factor homologous to human ATF2. Sty1 and Atf1 regulate a large set of core environmental stress response (CESR) genes in response to a range of stresses^2^. The fission yeast genome encodes three other ATF/CREB proteins: Atf21, Atf31 and Pcr1. Among these, *atf21* is specifically induced under conditions of high osmolarity^2^.

ncRNAs are transcripts that do not encode proteins. Although pervasively transcribed, the majority of ncRNAs have yet to be assigned a role, and their global functional significance remains controversial^3^. We previously identified a set of ncRNAs dependent on Atf21 (ref. 4). Studies of individual ncRNAs have revealed them to be a biochemically versatile class of molecules involved in a diversity of processes, including chromatin modulation, posttranscriptional regulation, the modulation of protein activity and the organization of protein complexes^5^. We therefore speculated that these ncRNAs might comprise additional effectors of the SAPK regulatory system.

Here we integrate high-throughput RNA sequencing and label-free quantitative protein mass spectrometry to investigate global changes in transcript and protein levels in the fission yeast stress response. These data reveal a genome-wide set of non-coding regions that together regulate protein levels throughout the cell. Deletion of one of these loci, *SPNCRNA.1164*, reduces the abundance of Atf1 and alters sensitivity to oxidative stress. These non-coding transcripts are therefore involved in regulating specific stress responses, and through association with Atf1, have the potential to add substantial information-processing capacity to this signalling system.

## Results

We combined strand-specific total RNA sequencing and label free tandem mass spectrometry to profile transcript and protein levels in wild-type (WT) and *atf21Δ* cells over a time course following addition of 1 M sorbitol. More than 76% of the (stranded) genome was transcribed, confirming previous reports^4^,^6^.

### Transcriptome and proteome changes under osmotic stress

Osmotic stress led to substantial reprogramming of gene expression in the WT cells. Three thousand one hundred and sixty-nine genes (out of 5,957) exhibited significant alterations (1,234 changed >2-fold at 5% false discovery rate (FDR)) in at least one time point (Supplementary Data 1). Deletion of *atf21* results in transcription profiles largely similar to that seen in the WT cells (Supplementary Fig. 1), with the total number of genes that changed significantly in at least one time point decreasing to 3,044 (1,079 changed >2-fold). Notably, 94 and 93% of the previously reported CESR genes^2^ were found to be differentially regulated in both WT and *atf21Δ* cells, respectively. Similarly, 17 out of 18 osmotic-stress-specific genes reported in ref. 2 were also called significant in both WT and *atf21Δ* time courses, suggesting that general osmotic stress responses of fission yeast were not impaired by *atf21* deletion.

In addition to CESR and osmotic stress-response genes, upregulated genes in WT and *atf21Δ* cells were significantly enriched for the following Gene Ontology (GO) Slim terms: carbohydrate metabolic process, ribosome biogenesis and protein catabolic process; downregulated genes were enriched for transmembrane transport and cellular amino acid metabolic process terms (Supplementary Fig. 2). Expression changes of genes encoding enzymes in lipid metabolism have been observed previously in *Saccharomyces cerevisiae* on osmotic shock^7^. Almost twice as many genes involved in lipid metabolic process were downregulated at 15 min in the WT than in the *atf21Δ* (46 in WT versus 26 in mutant), as are genes involved in the GO category ‘conjugation with cellular fusion’ (36 in WT versus 19 in mutant). Genes annotated to the functional categories DNA-dependent transcription and DNA replication were repressed for longer in *atf21Δ* mutant cells, suggesting a role for *atf21* in regulating these biological processes.

We next profiled protein abundance changes in the same samples using label-free quantitative protein mass spectrometry and compared changes in transcript abundance (as measured by the number of uniquely mapped reads aligning to the coding sequence (CDS) region) with corresponding protein level during osmotic stress. We detected and quantified 1,167 proteins in total, of which 417 (31 changed >2-fold) and 297 (32 changed >2-fold) exhibited significant variations in abundance in WT and *atf21Δ* mutant, respectively (FDR<0.05; Supplementary Data 2; Supplementary Fig. 3). Functional enrichment analysis showed that proteins involved in the stress response and carbohydrate metabolic processes were significantly upregulated, while those involved in ribosomal biogenesis and protein folding were downregulated (Supplementary Fig. 4).

Overall, transcript and protein abundance are dynamically coordinated during the osmotic stress time course. However, as opposed to the transient changes in transcript expressions, changes in protein abundance are more dramatic and persistent. For example, transcript expression of most upregulated genes transiently peaked at 30 min after stress induction and then decreased to steady-state level. The corresponding proteins, however, exhibited a delayed and gradual increase in abundance that maintained up to 180 min. Similar patterns of regulation have recently been reported in *S. cerevisiae* on osmotic stress induction^8^ and in *S. pombe* under various stress conditions^9^. Lee *et el.*^8^ proposed that the transient burst in transcript levels serves to accelerate protein change before mRNA levels acclimatize to new steady-state levels.

### Raised antisense RNA correlates with reduced protein levels

**Figure 1:**
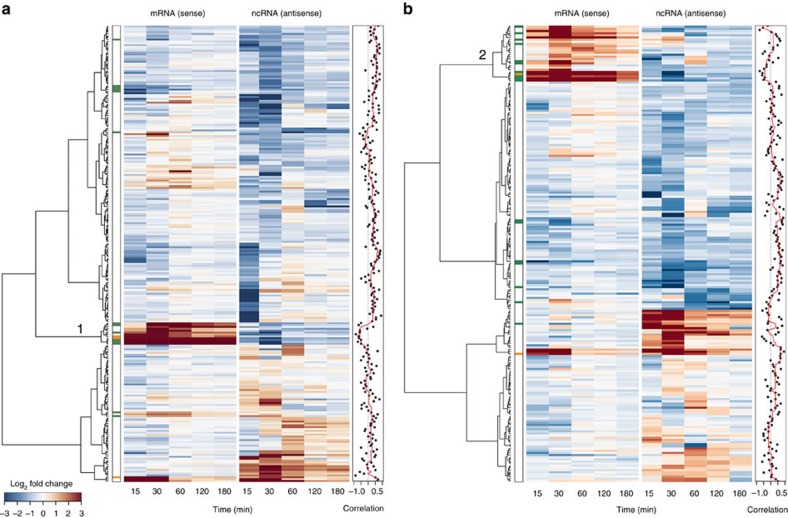
Stress-responsive ncRNA–mRNA sense–antisense pairs. (**a**) Sense–antisense pairs involving ncRNAs that changed significantly in abundance during osmotic shock in the WT. Rows represent log_2_ transcript levels (mean of three biological replicates) plotted relative to *t*=0, clustered by Euclidean distance. Columns are ordered according to timepoint. Pairwise Pearson’s correlation was computed for each pair (right hand panel). (**b**) Similar to **a**, but for the *atf21Δ* strain. Pairs in Clusters 1, 2 are anti-correlated in expression and functionally enriched for CESR (green) and osmotic stress-specific protein coding transcripts (orange).

**Figure 2:**
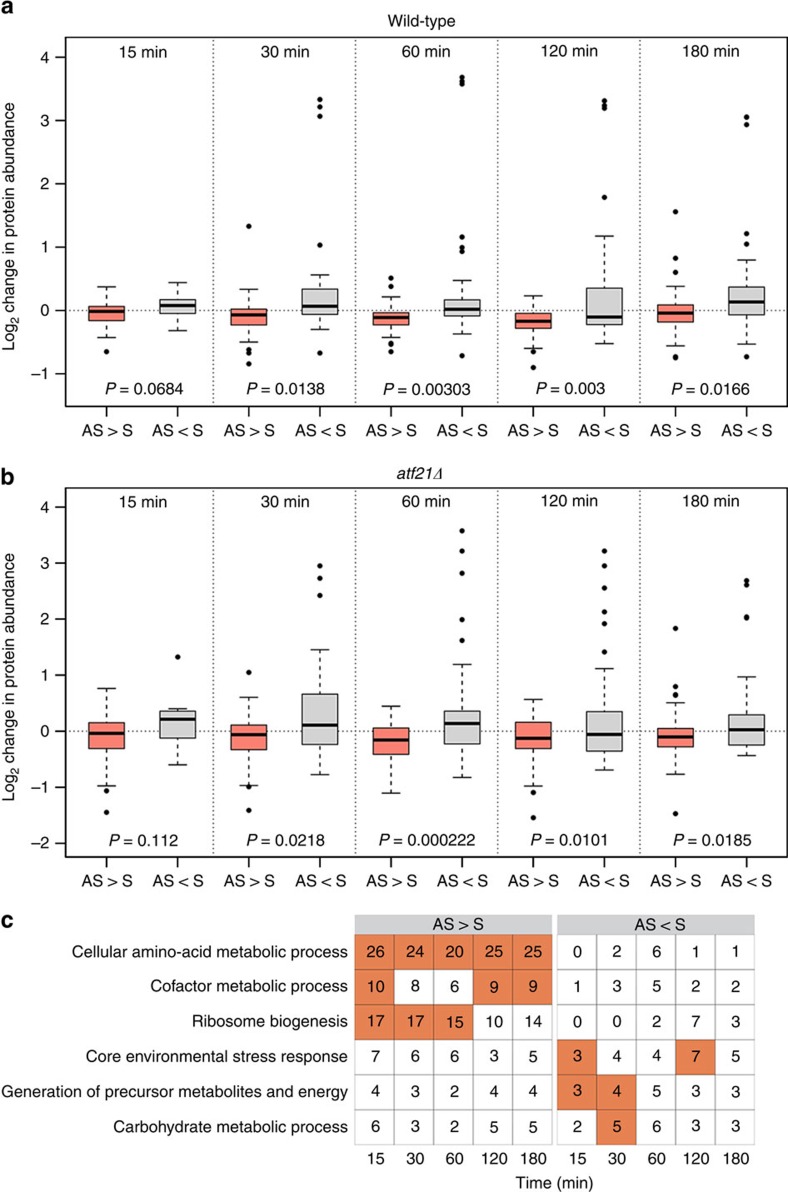
Global reduction of protein levels in the presence of *cis*-antisense. Box plots represent change in protein expression in WT (**a**) and *atf21Δ* (**b**) for which sense exceeds antisense (grey) and where antisense exceeds sense (red) in 140 sense–antisense pairs involving ncRNA. All data were normalized relative to *t*=0.Transcript levels are the mean of three biological replicates; protein expressions are the mean of five technical replicates. Whiskers correspond to the largest or smallest observation that falls within a distance of 1.5 times the interquartile range from the nearest hinge. *P*-value was calculated using *t*-test. (**c**) GO Slim categories that are significantly enriched in loci exhibiting either a higher level of antisense (AS>S) or of sense (S>AS) in **a** and **b**. The number of sense–antisense pairs for which the protein-coding transcripts were annotated to the GO Slim term is enumerated in each cell. Significant instances (hypergeometric test, FDR<5%) are coloured red.

Substantial shifts were also observed in ncRNAs. Two hundred and forty-eight (16%) and 232 (15%) of known ncRNAs changed significantly in the WT and *atf21Δ* strains, respectively (Supplementary Fig. 5). Many were antisense to a protein-coding gene, forming 246 sense–antisense pairs in the WT, 212 in *atf21Δ* (Fig. 1a,b; Supplementary Data 3). As regulatory antisense transcripts are critical in meiosis^4^,^10^,^11^ we asked whether they act more generally. We reasoned that if antisense transcripts had a function in stress, then those changing across the time course would tend to occur opposite CESR genes. This was indeed the case: the set of protein coding genes opposite stress-responsive antisense transcripts was enriched for CESR genes (WT: 14/70, *P*=0.003; *atf21Δ*: 18/70, *P*=4 × 10^−6^, hypergeometric test). Pearson’s correlation between the expression profiles of each antisense ncRNA and its respective overlapping sense mRNA identified a set of highly anti-correlated pairs (Fig. 1; clusters 1 and 2) that were, again, significantly enriched both for CESR genes (*P*=6 × 10^−7^, hypergeometric test) and those specific to osmotic stress (*P*=0.003). Antisense ncRNAs opposite these CESR genes tend to be downregulated. Mapping of the proteomics data to all sense–antisense pairs consisting of a protein-coding gene and an ncRNA revealed systematic reductions in protein levels at loci where antisense is in excess (Fig. 2a,b). A significant proportion of downregulated proteins are involved in amino acid metabolism, cofactor metabolism and ribosome biogenesis; categories with reduced antisense levels and increased protein production include the CESR and carbohydrate metabolism (Fig. 2c). These data implicate antisense ncRNAs in modulating protein production in response to stress.

### Global interactions between adjacent gene pairs

One thousand eight hundred and fifty-three sense–antisense pairs (60% mRNA:mRNA; 40% mRNA:ncRNA) resulted from transcription beyond the 5′- and 3′-ends of a CDS (Supplementary Data 4). We asked whether regulatory sense–antisense interactions could arise from these overlaps. *De novo* re-annotation of the 5′- and 3′-boundaries of each gene using sequencing data from the entire time course identified a substantial number of genes with boundaries extending significantly further than currently annotated^12^. We performed a systematic comparison of the 3′ extents of 3,655 genes that have unambiguous boundary assignment in our study to the untranslatedregion (UTR) lengths reported in (i) Mata *et al*.^13^ and (ii) PomBase (Supplementary Fig. 6a,b). Two different UTR assignments were considered in ref. 13, one based on the most distal cleavage and polyadenylation site identified using the 3′ PolyA site mapping using complementary DNA circularization) protocol, and a second, based on the peak of the most distal cluster. Overall, the 3′-end defined by our approach shows good agreement with PomBase’s UTR annotation (Pearson’s correlation=0.97) and the longest UTR defined using the 3′ PolyA site mapping using cDNA circularization technique (Pearson’s correlation=0.94), but less so to boundaries identified from the cluster peak (Pearson’s correlation=0.68). These data suggest that many of the extensions that we observed in our data are likely to be due to extended transcript UTRs. However, as our data were generated using total RNA, it is possible that some of these extensions were also due to unprocessed transcripts.

**Figure 3:**
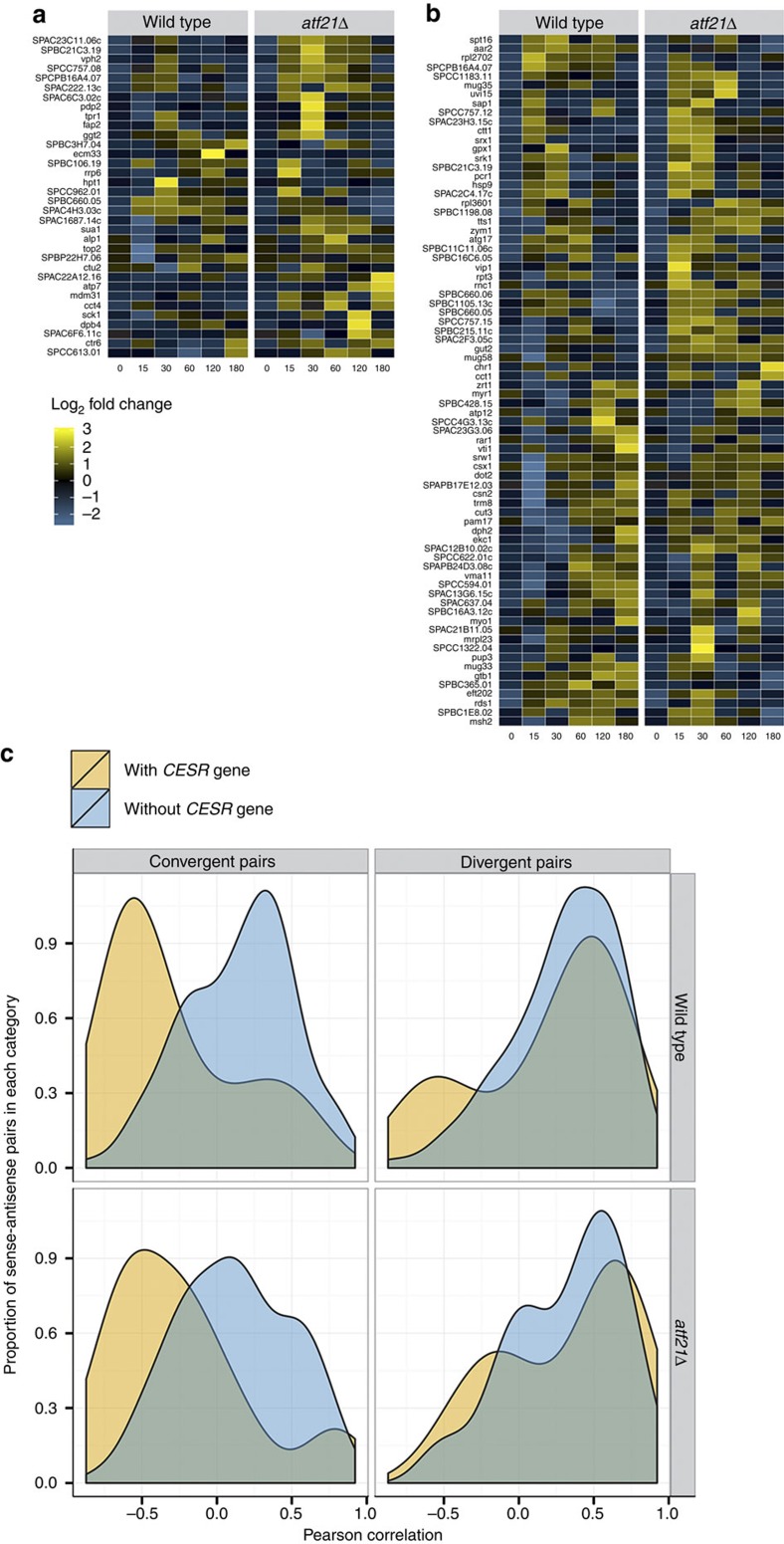
5′- and 3′-ends of genes are systematically regulated in response to osmotic stress. (**a**) Thirty-four loci exhibited significant alterations in the length of untranslated regions beyond the 5′-CDS boundary in response to osmotic stress (FDR<8%). Heatmaps are coloured by the fold change in length, relative to *t*=0 (log_2_ scale). Yellow indicates longer fold change, blue indicates shorter fold change. (**b**) Similar to **a**, but for the 76 genes with significant changes at the 3′-end. (**c**) Pearson’s correlation of transcript levels was calculated for the set of adjacent gene pairs (WT, *n*=246 pairs; *atf21Δ*, *n*=212 pairs) involving differentially expressed ncRNA transcribing towards each other (convergent pairs) and away from each other (divergent pairs). Convergent gene pairs involving a CESR gene (yellow) tend to be anti-correlated relative to those without a CESR gene (blue). Divergent gene pairs tend towards positive correlation. WT (top); *atf21Δ* (bottom).

The length of both the 5′- and 3′-non-coding regions segregated by GO Slim functional category (Supplementary Fig. 6c; for approach see refs 6, 14). The longest mean 5′-extensions were associated with the osmotic stress response, possibly as a consequence of regulation by the selection of alternate initiation sites. Thirty-four 5′- and seventy-six 3′-ends became elongated under stress (FDR<8%; Fig. 3a,b; Supplementary Data 5). No functional enrichment was observed for genes with extended 5′-boundaries, but the 3′-extended set was enriched for CESR genes (*P*=1.35 × 10^−11^, hypergeometric test). Fifty five per cent of genes that change at the 3′-end form convergent sense–antisense pairs with a neighbouring locus that also changed during osmotic stress (*P*=0.0045, hypergeometric test).

Overall, a global pattern was observed in which convergent gene pairs involving at least one CESR gene are predominantly anti-correlated, while divergent gene pairs are largely positively correlated, irrespective of whether the sense gene was a CESR gene or not. This effect was increased when only stress-responsive mRNA:ncRNA pairs were considered (Fig. 3c; Supplementary Data 3). Taken together, these data are indicative of a global system distributed throughout the genome in which antisense transcripts, including those that arise from dynamic changes at the 5′- and 3′-ends of genes, are used to regulate protein expression. The recent identification of substantial isoform complexity in the budding yeast genome^15^ raises the possibility that these phenomena may result at least in part from the dynamic regulation of constellations of multiple overlapping isoforms, rather than simply from changes to the UTRs of a single predominant transcript.

### *SPNCRNA.1164Δ* cells are resistant to oxidative stress

**Figure 4:**
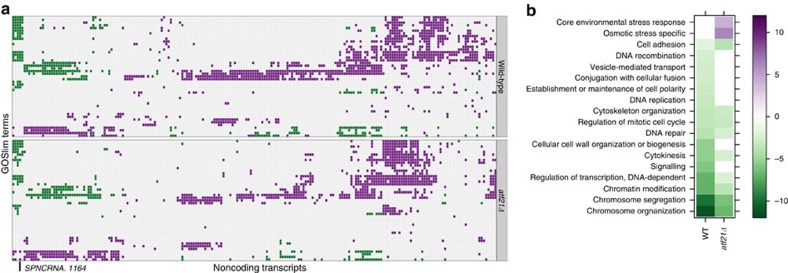
Significant associations between ncRNAs and protein coding gene families. (**a**) Stress-responsive ncRNAs are significantly associated with a diverse set of functionally related groups of genes. Coloured cells correspond to ncRNAs (columns) that are positively correlated (Pearson’s *ρ*>0.7; purple) or anti-correlated (Pearson’s *ρ*<−0.7; green) to the expression profiles of a significant number of protein-coding transcripts in a given GO Slim category (rows), at FDR<5%. Selected families are highlighted. Complete annotation for all differentially expressed ncRNAs in both WT and *atf21Δ* strains can be found in Supplementary Fig. 7. (**b**) Specific functional associations for *SPNCRNA.1164* in the WT and *atf21Δ* strains, as calculated for **a**; coloured using −log_10_ *P*-value.

**Figure 5:**
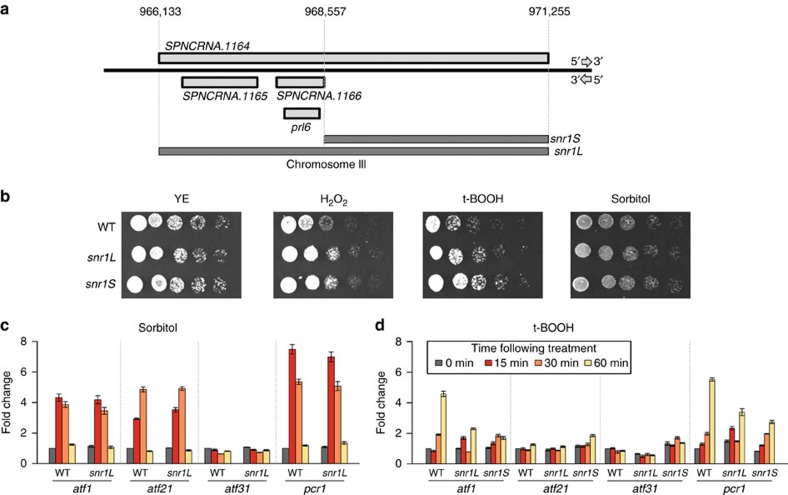
Osmotic stress-responsive ncRNA *SPNCRNA.1164* alters the oxidative stress response. (**a**) The *SPNCRNA.1164* locus, indicating the regions of the genome that were deleted. Two deletion strains were generated: (i) *snr1L*—the full-length transcript was deleted, and (ii) *snr1S*—the 5′-non-overlapping portion of the transcript independent of *SPNCRNA.1165* and *SPNCRNA.1166/prl6* was deleted. (**b**) Serial dilution assays reveal resistance to H_2_O_2_ (1 mM) and t-BOOH (0.75 mM), but not to sorbitol (1 M) in both *snr1L* and *snr1S* strains. The experiment was performed three times and representative results are shown. (**c**) Transcript levels of the four ATF transcription factors in the WT and *snr1L* cells following induction of stress by treatment with sorbitol (1 M) at indicated timepoints. (**d**) The four ATF transcription factors exhibited similar response to t-BOOH (0.75 mM) in both *snr1L* and *snr1S* strains. Mean and s.e.m. of three biological replicates are shown in **c** and **d**.

As *cis*-acting regulatory interactions between overlapping transcript pairs have been successfully recapitulated in *trans* from a spatially distinct locus^4^, we hypothesized that natural *trans*-acting non-coding regulators might also occur. We grouped protein-coding genes by GO Slim category, and then searched for ncRNAs with significantly correlated expression profiles to a disproportionate number of members of a given functional group. (|*ρ*|>0.7; hypergeometric test, FDR<5%). ncRNAs were found to be associated with 45 functional categories, including CESR, osmotic stress and carbohydrate metabolism (Fig. 4a; Supplementary Fig. 7; Supplementary Data 6). Although these associations were generally well preserved between the WT and *atf21Δ*, specific non-coding loci behaved differently. We pursued one further, *SPNCRNA.1164*, by phenotypic characterization of its deletion strain. This transcript was chosen because deletion of *atf21* substantially modified its associations with functional groups, including CESR- and osmotic stress-specific transcripts, and signalling (Fig. 4b). Serial dilution assays were used to monitor the sensitivity or resistance of the deletion strain to a panel of stresses (sorbitol, hydroxyurea, CdSO_4_, KCl, CaCl_2_, H_2_O_2_, tert-butyl hydroperoxide solution (t-BOOH)). Deletion of the full-length transcript (*snr1L*) led to mild resistance to H_2_O_2_ and t-BOOH, but had no effect in response either to sorbitol (Fig. 5b) or the other stresses tested (data not shown). Transcript levels of the four ATF transcription factors were maintained in response to sorbitol (Fig. 5c), but *atf1* and *pcr1* transcript levels were reduced significantly following treatment with t-BOOH (Fig. 5d). *SPNCRNA.1164* is complementary to two shorter antisense ncRNA regions within the locus: *SPNCRNA.1165* and *SPNCRNA.1166*/*prl6* (Fig. 5a). Deleting the 3′ non-overlapping portion of the gene (*snr1S*) recapitulates the phenotypes observed for *snr1L* under both oxidative and sorbitol stress (Fig. 5b). Levels of the four ATF transcription factors also behave similarly in response to t-BOOH (Fig. 5d).

### Loss of *SPNCRNA.1164* alters levels of Atf1

**Figure 6:**
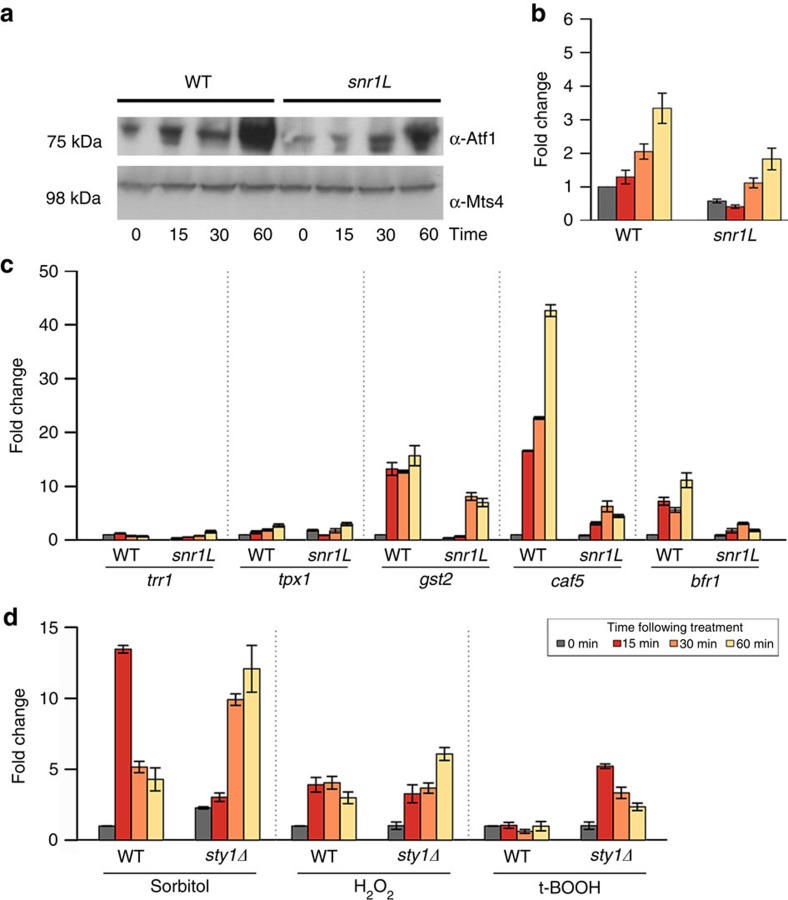
Atf1 protein levels are dependent on *SPNCRNA.1164*. (**a**) Representative western blotting showing decline in Atf1 protein levels in *snr1L* compared with WT cells following treatment with 0.75 mM t-BOOH. (**b**) Quantification of **a**. Mean and s.e.m. of five biological replicates are shown. (**c**) Transcript levels of known oxidative stress-responsive transcripts. (**d**) *SPNCRNA.1164* levels following induction of stress by treatment with sorbitol (1 M), H_2_O_2_ (1 mM) and t-BOOH (0.75 mM) in WT and *sty1Δ* cells. Mean and s.e.m. of three biological replicates are shown in **c** and **d**.

As Atf1 is a major downstream effector of the SAPK pathway^16^, we asked whether changes in the protein levels mirrored those of the transcript. Western blotting confirmed that a >3-fold reduction in protein levels were observed within 15 min following addition of t-BOOH (one-sided *t*-test, *P*=0.02; Fig. 6a,b). Levels of five transcripts known to be induced by oxidative stress (*trr1*, *tpx1*, *gst2*, *caf5*, *bfr1*) were monitored by quantitative PCR. The magnitude of induction for three transcripts (*gst2*, *caf5*, *bfr1*) was significantly reduced in the *snr1L* strain (Fig. 6c). We asked whether *SPNCRNA.1164* levels were dependent on the MAPK Sty1 upstream of Atf1. In WT cells *SPNCRNA.1164* was induced in sorbitol and H_2_O_2_ but not t-BOOH. Deletion of *sty1* substantially alters these dynamics (Fig. 6d). As it has been previously shown that *atf1Δ* cells are resistant to t-BOOH^17^, it is tempting to speculate that the *snr1Δ* phenotype observed here may, in part, be a consequence of reduced Atf1 levels. However, unlike *atf1Δ* cells, loss of *snr1* does not confer sensitivity to H_2_O_2_ (ref. 17), suggesting that precise Atf1 levels may be important, and that other factors are likely to be involved, such as the cell integrity pathway that signals into Atf1 via the MAPK Pmk1 (ref. 18).

## Discussion

Together, these results reveal a set of interlocking global systems comprising protein coding genes, regulatory overlaps between adjacent gene pairs and multiple independent ncRNAs. Examination of one locus reveals that these transcripts can have a direct effect on the level of Atf1, demonstrating for the first time that ncRNA systems have a role in the core stress response system, and adding further branches to the central MAPK signalling pathway. Their potential importance is further highlighted by the recent identification of a stress-induced ncRNA that acts as a positive regulator of Cdc28, the cyclin-dependent kinase 1 in budding yeast^19^. The stress response is complex and must be temporally staged, modulated according to the level of stress, programmed to cross-protect against future insults that are likely to occur^20^,^21^,^22^ and mounted at physiological timescales. It requires substantial levels of regulatory control. The addition of ncRNA to the systems that control Atf1 levels adds further complexity to a protein that acts not only as a transcription factor downstream of the SAPK pathway^16^, but also in the nucleation of constitutive heterochromatin^23^. The existence of multiple stress-dependent ncRNAs with strong statistical associations to protein coding loci (Fig. 4) suggests their involvement in many systems, although the precise mechanisms by which this is achieved have yet to be elucidated. Detailed studies have revealed a spectrum of related but different mechanisms by which ncRNAs can function^24^,^25^,^26^,^27^,^28^,^29^,^30^,^31^,^32^,^33^, suggesting that different loci are likely to be regulated through different combinations of mechanisms.

The ubiquity of non-coding genes, combined with the ability of cells to invoke rapid changes to the transcriptome, suggests that ncRNAs may lend considerable information processing capacity to the regulatory systems within a cell. The high conservation of core components of the chromatin modification, RNA interference and MAPK pathways between *S. pombe* and *Homo sapiens* suggests that similar mechanisms also exist in human cells.

## Methods

### Strains and stress experiments

*S. pombe* strains used in this study are listed in Supplementary Table 1. The deletion mutants were constructed by PCR deletion technique^34^ using pFA6kan^R^MX6 or pFA6natMX6 as templates^35^. Standard genetic procedures and culture methods of *S. pombe* were carried out following protocols in Moreno *et al*.^36^

For the stress experiments, cells were cultured in yeast extract medium and harvested immediately before and at different times (osmotic stress: 15, 30, 60, 120 and 180 min; all other stresses: 15, 30 and 60 min) after addition of stress-inducing agents to the culture. The stress conditions were induced by the addition of different drugs as follows. Osmotic stress: sorbitol (Sigma S6021) was added to a final concentration of 1 M. Genotoxic stress: hydroxyurea (Sigma H8627) was added to a final concentration of 2 mM. Heavy metal stress: cadmium sulphate (CdSO_4_, Sigma C2919) was added to a final concentration of 0.2 mM. Salinity stress: potassium chloride (KCl, VWR International Ltd 437025H) and calcium chloride (CaCl_2_, Sigma C3306) were added to a final concentration of 0.6 M and 100 mM, respectively. Oxidative stress: hydrogen peroxide (H_2_O_2_, Sigma H1009) was added to a final concentration of 1 mM; t-BOOH (70% in H_2_O, Sigma 458139) was added to a final concentration of 0.75 mM.

For the dilution assays, cells taken from an exponentially growing culture at a concentration of 1 × 10^6^ cells per ml were serially diluted five-fold a total of 4 times, 5 μl of each dilution including the starting dilution was spotted onto yeast extract plates containing no drug, 1 M sorbitol, 1 mM H_2_O_2_, 0.75 mM t-BOOH. The plates were incubated at 30 °C and photographed after 4 days.

### RNA extraction and library construction

Total RNA was extracted by treatment with hot acidic phenol–chloroform, followed by phenol–chloroform extraction and RNA precipitation. Detailed procedures can be found in ref. 37. Then RNA was column purified using QIAGEN RNeasy Kit 74104 according to the manufacturer’s instructions. RNA quality was determined using an Agilent 2100 Bioanalyser. Ribosomal reduction of total RNA (8 μg) was performed using the Ribominus Eukaryote Kit for RNA-Seq (Invitrogen A10837-08) and the Ribominus Concentration Module (Invitrogen K155005). More than 90% ribosomal reduction was achieved for all samples when assessed on the Agilent 2100 Bioanalyzer. Libraries were prepared with 350 ng of ‘ribosomally reduced’ total RNA using the SOLiD Total RNA-Seq Kit (Life Technologies 4445374). We used the chemical hydrolysis method to fragment the RNA and performed size selection on Novex 6% TBE-Urea Gels (Invitrogen EC6865). Samples were multiplexed by barcoding with the SOLiD Transcriptome Multiplexing Kit (Life Technologies 4427046). Library quantification was carried out with the Quant-IT DSDNA HS Assay Kit (Invitrogen Q32851) on the Qubit Fluorometer (Invitrogen Q3287). Molarities were determined using a High Sensitivity DNA Assay (Agilent Technologies 5067-1504) before pooling the libraries in equimolar quantities. Emulsion PCR was carried out on the pooled libraries using an input of 0.5 pM in the EZ Bead System to prepare template beads following the manufacturer’s instructions (Life Technologies PN 4448417). Templated beads were then sequenced on a 5500XL Genetic Analyser (Life Technologies 4460730) according to the manufacturer’s instructions, to generate 50 bases strand-specific reads.

### Quantitative real-time PCR

cDNA was made using the QuantiTect Reverse Transcription Kit (200) (Qiagen 205313). Real-time PCR was performed using the Roche Probe Library system and the JumpStart Taq ReadyMix (Sigma D6442-400RXN) on the Applied Biosystems 7900 machines. Data were analysed with SDS2.4 software (Applied Biosystems). Relative transcript expression was normalized to Cdc2 and Ptc2. All primers are listed in Supplementary Table 2.

### Sequencing data analysis

Over 3.5 billion single-end 48 nucleotide reads were obtained from all sequencing libraries. Sequencing reads were aligned to the fission yeast genome (PomBase database release 11) using SHRiMP2 (ref. 38) with default parameters. Total number of reads that can be aligned to the genome at exactly one locus per sample range from 7.5 to 20.1 millions. These uniquely mapped reads were used to identify stretches of unambiguous transcription. Reads that aligned to more than one locus (generally paralogous regions in the genome) were discarded. Adjacent unambiguous transcription regions with minimum peak height of 2 and located within 50 bases of each other were merged into transcribed loci to yield an extensive transcription map of *S. pombe*. These regions were then positioned relative to known annotation and labelled according to the gene(s) they overlapped, using the Bioconductor package annmap^39^. Next, we identified for each transcribed locus its 5′- and 3′-extent in a given sample. Subsequent analysis was performed in R and Bioconductor software^40^ unless otherwise stated.

### Expression measure

The expression of a locus was measured by the reads per kilobase per million mapped (RPKM) score, which was calculated by counting the number of uniquely mapped reads in a given locus and normalizing for by transcript length and for the total read count in the library prep^41^. Unless otherwise stated, the RPKM values were used to calculate the log-transformed fold change (base 2) for analyses described in the text.

### Identification of stress-responsive transcripts

The Bioconductor packages limma^42^ and edgeR^43^ were used to identify transcripts that showed statistically significant variation in abundance in the stressed cells relative to unstressed cells at 0 min time point. The raw counts, not the RPKM, were used for differential expression analysis in edgeR. First, we filter the data such that the only feature (or gene) with at least one count-per-million reads in more than six samples were kept. Next, the data were normalized with the TMM (trimmed mean and M values) method and separate generalized linear models were fitted for WT and *atf21Δ* samples using the respective unstressed samples as reference. A feature is considered as significant if it is differentially expressed in at least one time point relative to unstressed reference (0 min). Five different contrasts were formulated to ask what genes respond to osmotic stress at each time point. Multiple hypothesis testing correction was performed across all contrasts so that the overall FDR is kept below 5%.

### Functional enrichment analysis of non-coding transcripts

We used a correlation-based approach to classify the putative function of the stress-responsive ncRNAs. In brief, we correlated the expression profile of each ncRNA to the expression profiles in the CDS region of the protein-coding gene, and used a hypergeometric distribution-based over-representation analysis approach to determine whether any functionally related groups of genes are significantly enriched within the set of protein coding genes that exhibited high correlation (absolute Pearson’s correlation coefficient>0.7) with the candidate ncRNA. We used GO Slim terms (see below for details) to group the protein coding genes into gene families. Next, we constructed an association matrix between ncRNAs and the significant terms (FDR<5%). The resulting matrix was clustered using the Euclidean distance metric and complete linkage method.

### GO Slim and gene set collection

A list of 43 GO Slim terms^44^ specific for *S. pombe* genes was obtained using the online GO Slimmer tool (http://amigo.geneontology.org/cgi-bin/amigo/slimmer). Three additional gene sets were manually added to the collection, including ‘core environmental stress response’, ‘osmotic-stress specific’ and ‘non-coding RNA’. The ‘core environmental stress response’ gene set contains 178 CESR genes reported in ref. 2, the ‘osmotic stress-specific’ gene set contains 18 genes reported in 2 and the ‘non-coding RNA’ gene set contains 1,568 known non-coding transcripts from PomBase.

### Determining systematic change in gene boundaries

We used a permutation-based approach to systematically assess whether a gene has 5′- or 3′-boundary that is consistently longer in a specific time point following stress induction compared with that in the unstressed cells at 0 min. The extent of the boundary was defined as the distance from the first nucleotide in the 5′- or 3′-end to its CDS. We used the aligned RNA-Seq data to re-map all gene boundaries in the genome and filtered the data using the following criteria: (1) loci with boundaries spanning multiple open reading frames were discarded, (2) genes with mean expression (as measured by log_2_ RPKM) in the coding sequence region <2 were removed because these loci typically have low number of reads and thus unreliable boundary determination, (3) boundary data are missing when a gene is not expressed or does not have reads aligned to the re-mapped untranslated region. We retained only the genes for which boundary data is available in at least two of the three replicates per stress time point. After these filtering steps, 2,567 (5′-ends) and 3,036 (3′-ends) genes remained. For each gene we computed a metric, denoted as *L*, to estimate the range that we would expect to see the boundary vary. *L*=[mean (*y*)−2 × s.d.(*y*), mean (*y*)+2 × s.d.(*y*)], where *y* are pooled boundary extents at 0 min from the WT and mutant time courses. We then checked whether the boundary extents detected at 15, 30, 60, 120 and 180 min following stress induction fall within or beyond the range given by *L*. Ten thousand permutations were performed by randomizing the sample labels across the entire osmotic stress time course to assign significance (empirical *P*-value) to the observed magnitude of change. For each gene boundary, the empirical *P*-value was calculated as the proportion of times the boundary extent in stress conditions was longer than that in the unstressed cells at 0 min in the randomized samples. We considered boundaries that changed by least 30 nucleotides in all three replicates in a specific time point relative to 0 min with empirical *P*-value<0.05 as significant (FDR<8%).

### Label-free quantitative proteomics

#### Protein reduction, alkylation and digestion

A total of 50 μg of protein extract was reduced by addition of TCEP (tris(2-carboxyethyl)phosphine) to a final concentration of 5 mM at 60 °C for 60 min. Reduced cysteine residues were then alkylated with the addition of methyl methanethiosulphonate to a final concentration of 10 mM followed by 10 min incubation at room temperature. Fifty micrograms of protein was then digested by addition of 5 μg of sequencing grade trypsin followed by agitated incubation at 37 °C for 18 h as previously described^45^. The digests were desalted as previously described by Villen and Gygi^46^ as follows. The total 50-μg peptide digest was loaded onto a SepPak C18 SPE cartridge in 1 ml of 0.4% (v/v) TFA (trifluoroacetic acid) and peptides were desalted by addition of 5 ml 0.1% (v/v) TFA followed by 1 ml 0.5% (v/v) HAcO (acetic acid). Peptides were eluted by addition of 5 ml 50% (v/v) MeCN (acetonitrile), 0.5% HAcO. Desalted peptide digests were lyophilized to completeness before resuspension in 0.05% (v/v) TFA at a peptide concentration of 750 ng μl^−1^ for subsequent nano liquid chromatography–tandem mass spectrometry (MS/MS) analysis.

#### Nano liquid chromatography–MS/MS analysis

Seven hundred and fifty nanograms of peptides were separated using an RSLCnano HPLC (Dionex) as detailed below. Each sample was loaded onto an Acclaim PepMap C18 Nano-Trap column (300 μm internal diameter × 2-cm long, 5-μm particle size) in water, 1% (v/v) acetonitrile and 0.05% (v/v) triflouroacetic acid at a flow rate of 8 μl min^−1^ for 5 min. Peptides were then separated using an Acclaim PepMap C18 column (75 μm internal diameter × 100-cm long, 3-μm particle size) with a gradient of 1–35% (v/v) of acetonitrile, 0.1% formic acid over 70 min at a flow rate of 250 nl min^−1^. The nano-liquid chromatography effluent was sprayed directly into the LTQ-Orbitrap XL mass spectrometer (Thermo Fisher Scientific) aided by the Proxeon nano source at a voltage offset of 1.7 kV. The mass spectrometer was operated in parallel data-dependent mode where the MS survey scan was performed at a nominal resolution of 60,000 (at mass/charge (*m*/*z*) 400) in the Orbitrap analyser in an *m*/*z* range of 400–2,000. The top three precursors with charge states of 2, 3 and 4 were selected for collision-induced dissociation in the LTQ at a normalized collision energy of 35%. MS2 gas phase fractionation was employed to reduce redundancy within the data set. Each sample was analysed five times; in each case the MS1 data was collected in an *m*/z range of 400–2,000 and MS/MS acquisition was permitted only for multiply charged ions in the *m*/*z* range cuts of 400–525, 525–638, 638–766, 766–963 and 963–2,000. These gas phase fraction windows were calculated empirically from a pooled pilot injection to ensure each *m*/*z* window contained equal numbers of ions with charges considered for MS/MS. Dynamic exclusion was enabled to prevent the selection of a formally targeted ion for a total of 20 s.

#### MS data analysis

Following MS data acquisition, the Thermo Xcalibur RAW files were imported into Progenesis LC-MS software (version 3.0; Nonlinear Dynamics) with automatic feature detection enabled. A representative reference run was selected to which all other runs were automatically aligned in a pair-wise fashion. Features (peaks) were filtered for charge state (2, 3 and 4 accepted) and elution time window. The resulting MS/MS peak lists were exported as a single Mascot generic file and loaded onto a local Mascot Server (version 2.3.0; Matrix Science). We searched these spectra against the SwissProt/UniProt 2011 database (selected for *S. pombe*, X sequences, Y entries) using the following parameters: tryptic enzyme digestion with one missed cleavage allowed, precursor mass tolerance of 10 p.p.m., fragment mass tolerance of 0.6 Da, oxidation of methionines and deamidation of glutamine/asparagine as variable modifications, ion score significance threshold of *P*≤0.05. The resulting peptides were exported as an XML file from Mascot and imported back into Progenesis LC-MS to assign peptides to features. A table of all identified features along with their normalized peptide abundance in each sample was generated. In total, 7,330 (out of 58,211) features have peptide assignments and these peptides belong to 1,167 non-redundant proteins. We calculated an abundance measure for each protein by averaging the corresponding normalized peptide abundance. This results in a matrix consisting of 1,167 proteins and their respective abundance in each sample 5 technical replicate injections were available for each treatment, no biological replicate). This was analysed in the R/BioConductor package limma^42^ to identify stress-responsive proteins. A protein was considered as significant if its abundance showed differential changes in the stressed cells relative to unstressed cells in at least one time point at 5% FDR.

## Author contributions

H.S.L. performed the bioinformatics analyses. C.R.M.W. prepared samples of RNA and protein from *S. pombe.* K.D. conducted the molecular biology experiments. H.S.L., C.W., Y.L. performed initial alignment and data processing. Y.C., D.S. conducted the mass spectrometry. All authors planned the experiments and contributed to writing the manuscript.

## Additional information

**Accession codes:** Mass spectrometry data have been deposited to the ProteomeXchange Consortium (http://proteomecentral.proteomexchange.org) via the PRIDE partner repository (dataset identifier PXD000894; DOI 10.6019/PXD000894). RNA Sequencing data have been deposited to GEO (Accession code GSE56761).

**How to cite this article**: Leong, H.S. *et al*. A global non-coding RNA system modulates fission yeast protein levels in response to stress. *Nat. Commun.* 5:3947 doi: 10.1038/ncomms4947 (2014).

## Supplementary Material

Supplementary Information Supplementary Figures 1-7 and Supplementary Tables 1-2

Supplementary Data 1 Transcripts that exhibit statistically significant changes in response to osmotic stress in wild type and Atf21 mutant cells.

Supplementary Data 2 Proteins that exhibit statistically significant changes in response to osmotic stress in wild type and Atf21 mutant cells.

Supplementary Data 3 Sense-antisense pairs involving stress-responsive ncRNAs in wild type and Atf21 mutant cells.

Supplementary Data 4 Sense-antisense pairs involving overlapping gene boundaries involving at least one protein coding locus.

Supplementary Data 5 5′ and 3′ ends that displayed significant changes in length relative to 0 min during osmotic stress. Separate worksheets also record all estimated 5′ and 3′ ends across the genome.

Supplementary Data 6 Functional categories significantly associated with stress-responsive ncRNAs for wild type and Atf21 mutant cells.
