# Supplementary material for: A global non-coding RNA system modulates fission yeast protein levels in response to stress
Source: Nat Commun. 2014 May 23;5:3947. doi: 10.1038/ncomms4947 (PMC4050258; doi:10.1038/ncomms4947)
Supplement: Supplementary Information — Supplementary Figures 1-7 and Supplementary Tables 1-2 [file ncomms4947-s1.pdf]

Supplementary Fig. 1

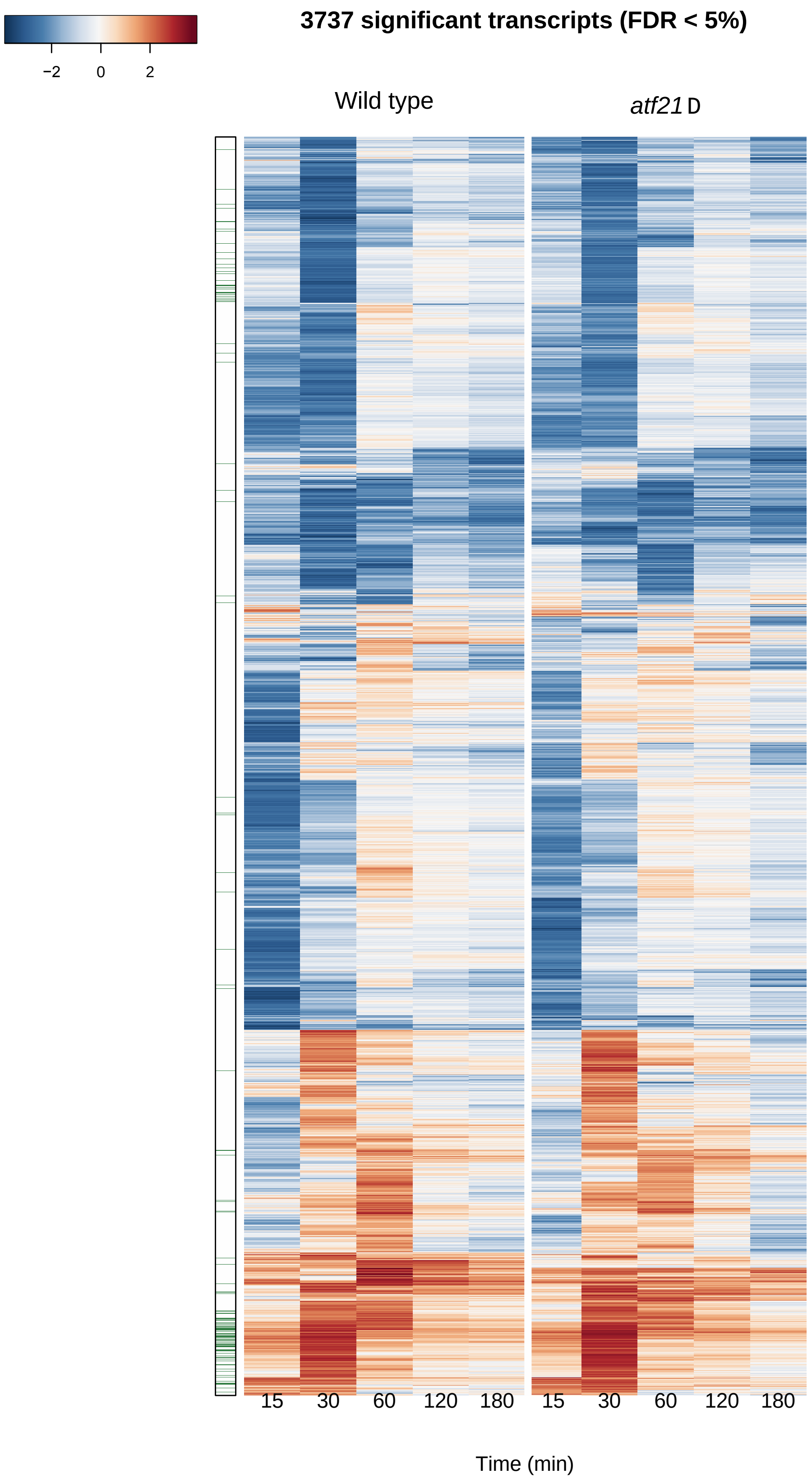

Supplementary Fig. 1 Global transcriptomic changes during osmotic stress

3,737 transcripts that changed significantly in abundance (FDR < 5%) in the wild type and *atf21Δ* strains during osmotic stress. Rows represent $\log_2$ transcript levels relative to 0 min, hierarchically clustered by Euclidean distance. Columns are ordered according to timepoint (three biological replicates per timepoint). Core environmental stress response (CESR) genes are indicated by green colored bar on the left.

Supplementary Fig. 2

	Wild type Up−regulation					Wild type Down−regulation					*atf21*Δ Up−regulation					*atf21*Δ Down−regulation				
Time (min)	15	30	60	120	180	15	30	60	120	180	15	30	60	120	180	15	30	60	120	180
vitamin metabolic process	3	8	7	3	2	1	4	8	4	5	7	7	4	3	1	1	4	9	5	4
cofactor metabolic process	26	29	20	7	7	8	15	20	14	14	34	34	22	7	7	6	20	19	15	9
vacuole organization	10	19	4	3	5	8	3	1	0	0	9	18	6	3	3	3	1	0	0	0
protein folding	19	14	15	1	1	2	10	14	10	9	11	13	12	0	0	6	13	12	11	7
osmotic stress specific	16	14	9	9	10	0	0	0	0	0	15	14	10	10	8	0	1	0	0	0
peroxisome organization	2	5	2	0	0	8	4	1	0	0	2	4	1	0	0	4	4	1	0	0
nitrogen cycle metabolic process	0	0	0	0	0	2	3	2	4	3	2	0	1	0	0	2	4	2	5	4
transmembrane transport	30	55	25	7	11	72	50	39	30	18	32	65	31	15	16	51	50	33	31	17
establishment or maintenance of cell polarity	8	21	6	3	3	20	10	0	0	1	8	20	6	3	2	16	8	0	0	0
generation of precursor metabolites and energy	19	28	16	8	7	6	8	11	10	7	26	31	18	10	7	2	8	10	9	3
carbohydrate metabolic process	30	59	42	14	14	24	21	8	5	8	36	64	44	28	19	18	16	9	6	5
core environmental stress response	85	104	90	63	67	17	50	39	8	6	89	103	84	68	50	14	49	31	9	1
protein modification by small protein conjugation or removal	26	59	24	2	3	24	21	3	1	2	18	58	23	6	5	14	19	0	1	0
protein catabolic process	45	85	45	2	4	23	13	3	5	3	39	81	38	4	3	13	12	3	4	2
ribosome biogenesis	42	7	4	1	1	13	179	97	1	3	43	6	3	2	4	17	185	105	2	2
cytoplasmic translation	66	10	7	1	1	10	53	141	10	13	122	12	9	1	4	7	41	131	15	4
nucleobase−containing small molecule metabolic process	58	86	42	8	10	61	87	52	17	22	64	84	50	12	8	49	97	48	19	11
tRNA metabolic process	10	4	3	0	1	12	57	33	5	7	14	3	1	1	0	12	72	30	9	2
nucleocytoplasmic transport	17	8	4	2	1	18	39	18	0	0	15	9	4	1	4	13	42	16	0	0
cellular amino acid metabolic process	37	25	18	5	5	13	40	77	42	46	53	30	15	8	4	10	38	69	53	28
non−coding RNA	39	57	31	6	8	81	73	59	34	32	46	72	29	12	4	36	74	64	42	33
cytokinesis	15	27	12	4	6	41	19	5	1	1	15	25	15	7	7	32	19	6	0	0
cell adhesion	0	3	2	2	2	10	3	2	0	0	0	1	4	2	2	10	4	2	0	0
protein glycosylation	3	4	3	0	0	19	10	1	1	0	1	6	5	1	1	10	11	0	1	0
transcription, DNA−dependent	11	30	10	2	3	30	35	17	1	0	6	24	10	1	0	24	53	17	3	0
DNA replication	9	16	4	3	3	37	26	5	3	2	5	15	4	4	2	30	35	4	3	2
DNA repair	9	28	7	4	2	45	23	3	0	1	2	16	3	4	0	34	28	3	2	3
cytoskeleton organization	24	31	13	6	6	46	20	14	0	3	24	33	16	4	4	37	35	11	1	2
lipid metabolic process	19	42	23	6	5	46	24	19	12	8	23	44	24	11	8	25	25	15	14	5
DNA recombination	5	11	1	1	1	26	13	2	0	0	4	10	2	3	0	26	19	0	1	2
chromosome segregation	10	20	4	2	0	56	30	8	3	4	9	19	6	1	2	43	46	6	2	3
chromosome organization	15	24	8	3	0	70	34	8	5	5	12	22	7	3	3	55	54	7	4	6
chromatin modification	12	31	8	1	0	46	30	7	1	1	8	29	10	1	2	48	45	10	6	3
regulation of transcription, DNA−dependent	31	74	24	10	12	83	38	14	5	3	22	62	27	11	10	70	56	14	10	7
meiosis	19	41	6	0	1	41	20	6	3	1	15	39	6	1	1	39	31	5	2	3
vesicle−mediated transport	32	74	25	5	7	66	32	7	4	2	37	77	32	9	6	48	39	5	4	3
conjugation with cellular fusion	10	26	11	4	5	36	12	8	1	1	9	25	15	5	6	19	14	4	2	2
signaling	39	88	25	10	17	78	30	15	3	3	39	82	32	12	17	53	38	17	4	1
regulation of mitotic cell cycle	44	78	34	11	10	58	33	9	4	5	38	71	24	13	9	48	42	7	5	6

Time (min)

Supplementary Fig. 2 Functional enrichment analysis of differentially regulated transcripts in response to osmotic stress

Gene Ontology (GO) Slim terms that are significantly enriched in the up- and down-regulated genes following sorbitol treatment in the wild type and *atf21Δ* cells are shown (hypergeometric test, FDR < 0.05). The count in each cell represents the number of differentially expressed genes that are annotated to the GO Slim term. Up-regulated genes that are significantly enriched for a specific functional term are colored in red, down-regulated genes in blue.

Supplementary Fig. 3

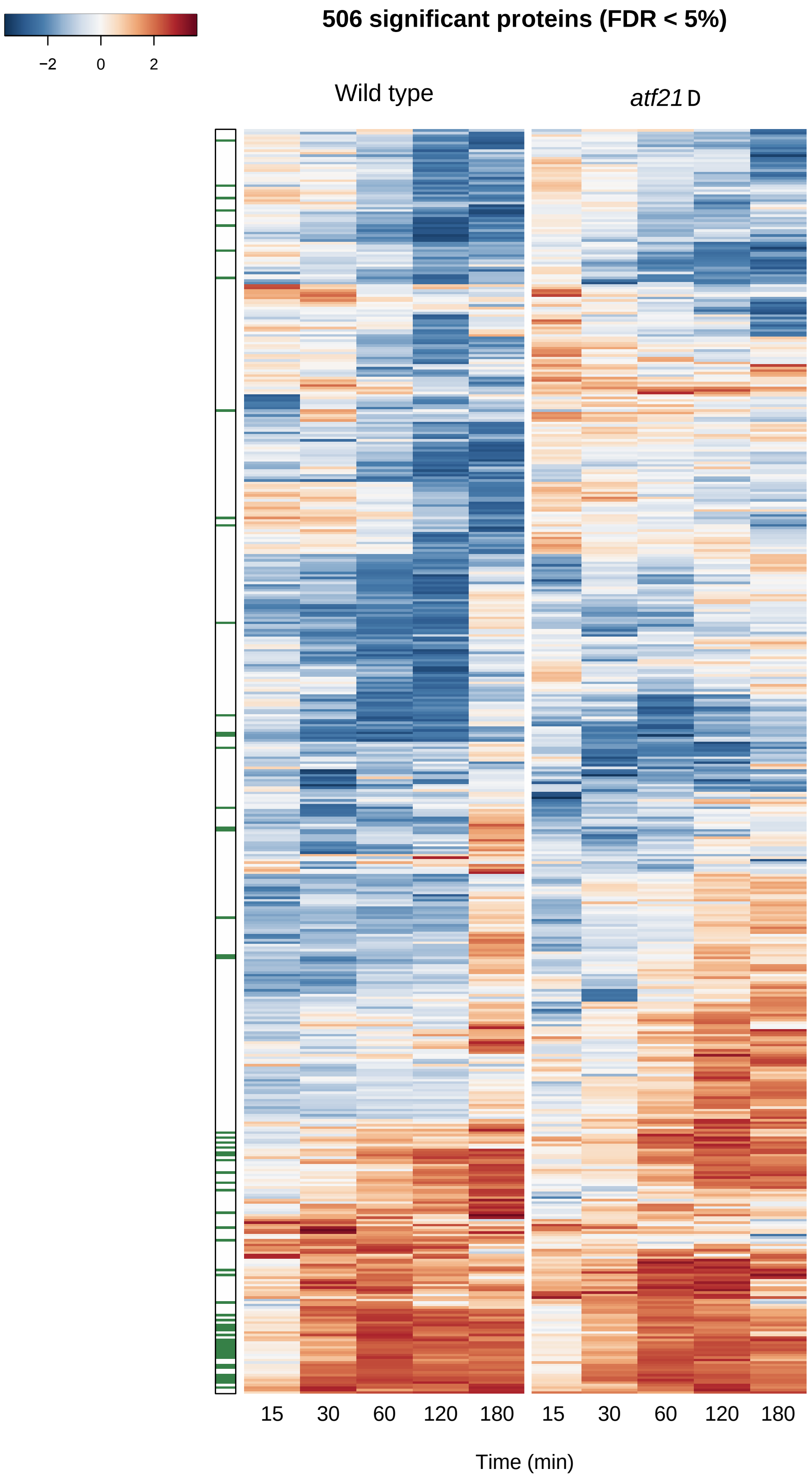

Supplementary Fig. 3 Changes in proteome during osmotic stress

506 proteins that changed significantly in abundance (FDR < 5%) in the wild type and *atf21Δ* strains during osmotic stress. Rows represent log_2 abundance levels relative to 0 min, hierarchically clustered by Euclidean distance. Columns are ordered according to timepoint (three biological replicates per timepoint). Core environmental stress response (CESR) genes are indicated by green colored bar on the left.

Supplementary Fig. 4

	Wild type Up−regulation					Wild type Down−regulation					*atf21*D Up−regulation					*atf21*D Down−regulation				
Time (min)	15	30	60	120	180	15	30	60	120	180	15	30	60	120	180	15	30	60	120	180
tRNA metabolic process	0	0	0	0	1	6	8	9	19	7	0	0	1	1	4	4	3	4	3	3
cytoplasmic translation	2	0	1	1	9	23	18	30	48	15	0	1	3	5	13	17	0	4	5	6
transmembrane transport	0	2	2	2	3	5	8	11	15	10	0	1	2	2	4	0	10	7	7	7
ribosome biogenesis	0	2	1	1	13	14	18	25	28	2	1	0	3	9	8	18	7	8	4	1
protein folding	1	1	0	0	1	4	3	6	16	11	1	1	1	0	1	1	1	1	5	13
cellular amino acid metabolic process	3	2	2	2	4	6	6	12	40	35	3	0	2	4	7	5	1	3	6	13
osmotic stress specific	2	2	2	2	2	0	0	0	0	0	1	1	2	1	1	0	0	0	0	0
protein glycosylation	0	0	0	0	0	2	3	0	0	0	0	0	0	0	3	0	4	1	0	0
core environmental stress response	10	27	32	30	32	4	8	10	12	9	4	22	31	32	32	3	5	5	8	6
carbohydrate metabolic process	4	13	9	9	11	1	1	2	5	8	2	6	10	13	9	0	1	1	2	4

Supplementary Fig. 4 Functional enrichment analysis of differentially regulated proteins in response to osmotic stress

As in Supplementary Fig. 1, but for differentially regulated proteins that showed statistically significant changes in abundance upon sorbitol treatment.

Supplementary Fig. 5

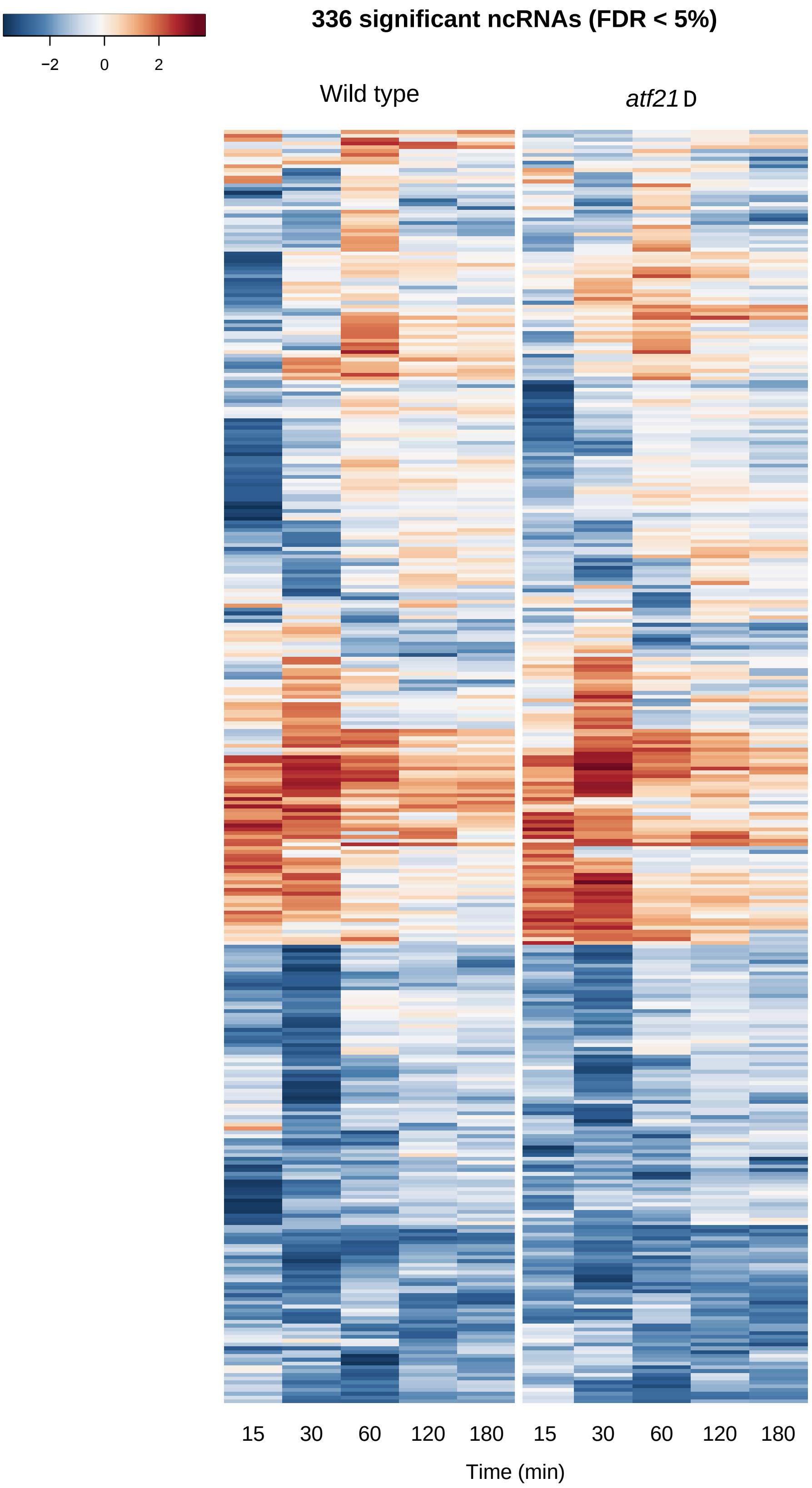

Supplementary Fig. 5 Osmotic stress induced changes in ncRNAs levels

336 ncRNAs that changed significantly (FDR < 5%) in the wild type and *atf21Δ* cells during osmotic stress. Rows represent $\log_2$ abundance levels relative to 0 min, hierarchically clustered by Euclidean distance. Columns are ordered according to timepoint (three biological replicates per timepoint).

Supplementary Fig. 6

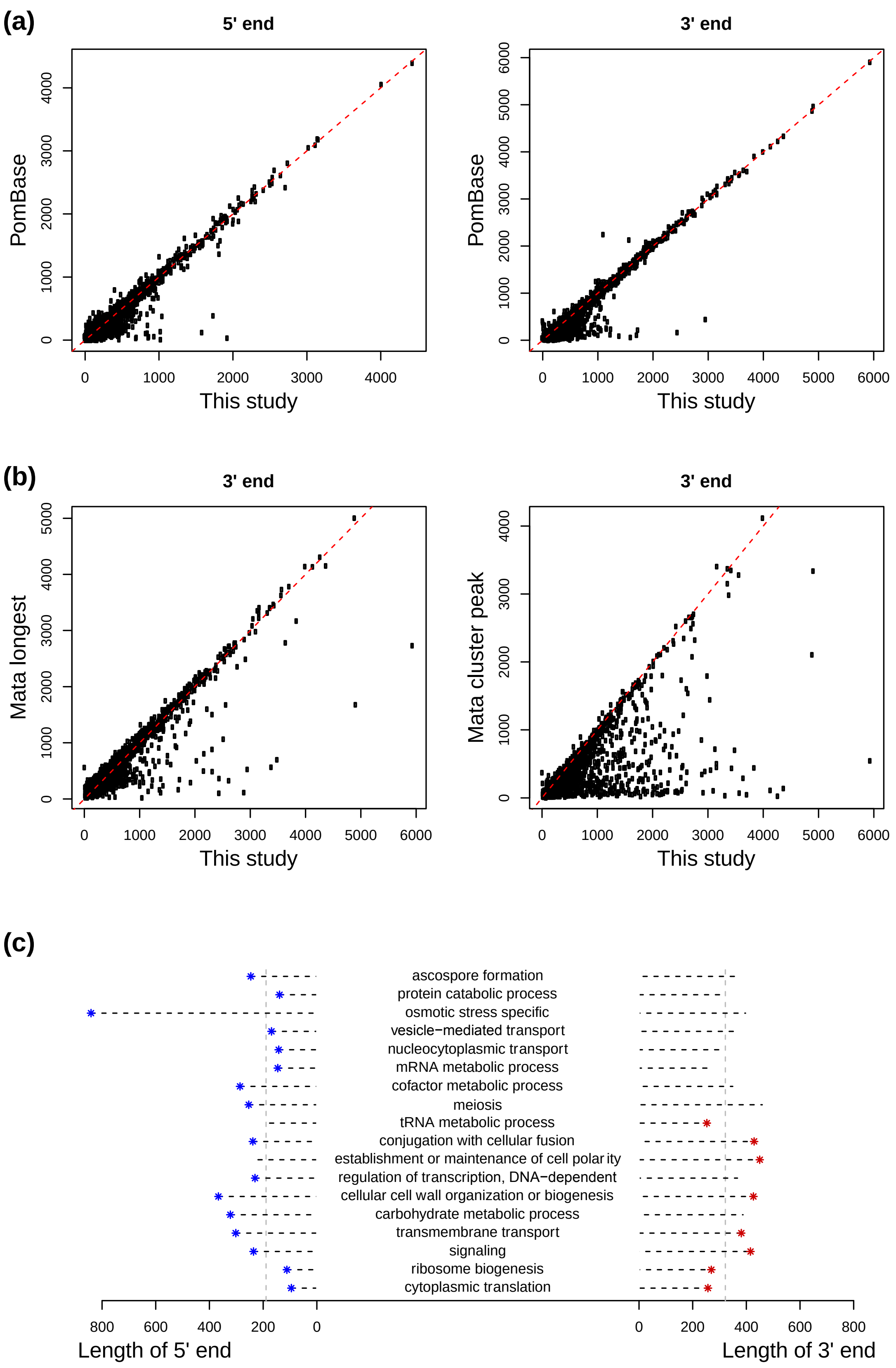

Supplementary Fig. 6 Context specific re-annotation of gene boundaries

The RNA-seq data of the entire osmotic stress time course encompassing both wild type and *atf21Δ* were used to perform a context specific re-annotation of the gene boundaries for each sample independently. For each gene the most distal 5′ and 3′ end was identified and used to define the maximal extent of the locus. (**a)** The lengths of these *de novo* re-annotations were compared to the length of annotated untranslated regions (UTR) in PomBase. **(b)** A comparison of the 3′ extents of 3655 genes that have unambiguous boundary assignment in our study to the two different UTR assignments reported in Mata *et al.* (2013, *RNA Biology*, 10: 1407-1414). The first assignment 'Mata longest' is based on the most distal cleavage and polyadenylation site identified using the 3PC (3′ PolyA site mapping using cDNA circularization) protocol, and the second assignment 'Mata cluster peak' is based on the peak of the most distal cluster. **(c)** Association between gene boundaries and GO Slim terms. Each row corresponds to a GO Slim category. The vertical grey dashed lines indicate median width of the boundary extents. Red (3′ end) and blue (5′ end) dots identify cases where the median for the category is significantly different from the genome-wide UTR median (two-sided Wilcoxon test, FDR < 5%).

Supplementary Fig. 7

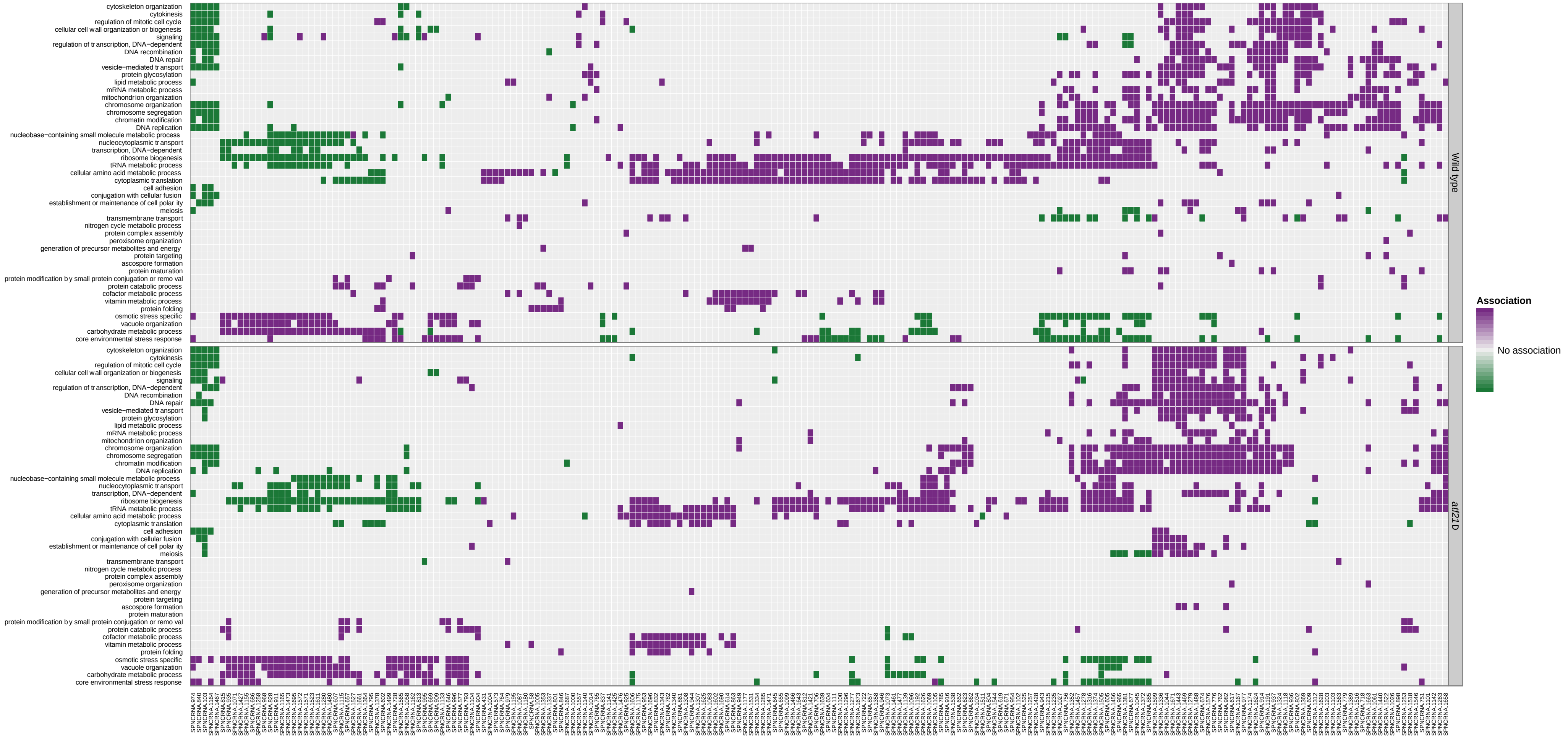

Supplementary Fig. 7 Significant associations between ncRNAs and protein coding gene families in the wild type and *atf21Δ* cells

Functionally related groups of genes that were significantly associated with the osmotic stress-responsive ncRNAs in the WT and *atf21Δ* cells. Colored cells correspond to ncRNAs that are positively correlated (Pearson $\rho > 0.7$; purple) or anti-correlated (Pearson $\rho < -0.7$; green) with expression profiles of a significant number of protein coding transcripts in a given GO Slim category (FDR < 5%).

Supplementary Table 1. ***S. pombe* strains used in this study**

Strain	Genotype	Source
**IH5974**	*972h⁻*	Stock from Iain Hagan's lab
**IH8810**	*atf21::kanMX6 ade6.M210*	Stock from Iain Hagan's lab
**NJ2**	*h⁺ ura4-D18 leu1-32 ade6-M210 his7-366*	Stock from Nic Jones's lab
**NJ293**	*sty1::kan ura4-D18 leu1-32 ade6-M210 his7-366*	Stock from Nic Jones's lab
**CJM74**	*972h⁻ srn1L::natMX6*	This study[1]
**CJM84**	*972h⁻ srn1S::natMX6*	This study[2]

[1] In this deletion strain, the full-length *SPNCRNA.1164* transcript located at chr III: 966,133-971,255 (genomic coordinates are based on Pombase database release 11) was deleted using the PCR deletion technique described in Online Methods. This deletion strain is referred to as Stress-responsive Non-coding RNA 1 Long *(snr1L)* in the paper.

[2] In this deletion strain, only the 5′ fragment of *SPNCRNA.1164* (chr III: 968,557-971,255), which does not overlap with other non-coding transcripts, was deleted. This deletion strain is referred to as Stress-responsive Non-coding RNA 1 Short *(snr1S)* in the paper.

Supplementary Table 2. Primers used in this study

ID	Forward primer	Reverse primer	[a]Probe #
Atf1	ttccatagcatcacctgatatctt	ccaagcaggatagcccaac	1
Atf21	tgcgaaggaattcgacaata	tcaatgtttctgaaaggacataattc	110
Atf31	cgaggcttcacacaactcac	ttggaagtgttttcatcacctg	42
Pcr1	tccgatgatgtccagcaa	cccaccaagggatctagga	66
SPNCRNA.1164	tctttctttttctcttctctattgctc	caattcaatcaatttgggttca	153
Sty1	tctttgatcctcgtaagcgtatt	ggatcatggtatggagcaaga	146
Caf5	cgtcagtacgctcttttacgc	gaagcagccaaatactgaaaca	57
Bfr1	cccgaaaaagacaactttgg	gacgatagagctcatgcttgg	53
Tpx1	gacgaggctctccgtcttc	gcaaacctcaccgtgctc	97
Gst2	aaagcttacgcttggaatcaa	tgccaattcttcgaaagtagc	62
Trr1	ttttggctactggtgcttcc	tgccaataagtatcctcaccagt	141
Cdc2	aaaaatacatggaccgaatttca	ttttgaacaagtctcggatctaaac	57
Ptc2	ggctgcagacaatgcgtta	gtagctgtgcatcccgaag	4

[a]This is the probe number from Roche Applied Science’s Universal ProbeLibrary.
